# Gut Microbiota Is the Key to the Antidepressant Effect of Chaihu-Shu-Gan-San

**DOI:** 10.3390/metabo10020063

**Published:** 2020-02-10

**Authors:** Meng Yu, Hong-Mei Jia, Tao Zhang, Hai Shang, Hong-Wu Zhang, Li-Yan Ma, Zhong-Mei Zou

**Affiliations:** Institute of Medicinal Plant Development, Chinese Academy of Medical Sciences and Peking Union Medical College, Beijing 100193, China; yumeng.5555@163.com (M.Y.);

**Keywords:** CSGS, antidepressant, antibiotic treatment, gut microbiota, metabolomics

## Abstract

Accumulating evidence highlights the link between gut microbiota and depression. As an antidepressant herbal drug in clinic, Chaihu-Shu-Gan-San (CSGS) has also been used in China for the treatment of various gastrointestinal disorders. Therefore, we hypothesize that the gut microbiota might be involved in the effect of CSGS. Here, we investigated the antidepressant effects of CSGS against chronic variable stress (CVS)-induced depression rats with and without antibiotic treatment using 16S rRNA gene sequencing and ultra-performance liquid chromatography coupled with time of flight mass spectrometry (UPLC-Q-TOF/MS) based metabolomics approaches. As a result, the prominent effects of CSGS against the depression-like behavioral disorder of CVS-induced rats were significantly weakened when the gut microbiota was changed after oral administration of the broad-spectrum antibiotic. The mediation of CSGS on hippocampal levels of serotonin (5-HT) and glutamic acid (Glu) was also receded with the antibiotic treatment. Further investigation on the diversity of microbiome indicated that the improvement effect of CSGS on gut microbiota dysbiosis—especially the phylum level of *Firmicutes—*was attenuated after the CSGS combined antibiotic treatment. Moreover, 3-hydroxypicolinic acid (H4) and inosine (H8) in the hippocampus were considered as important biomarkers for depression and are also associated with gut microbiota mediated CSGS efficacy. Taken together, our current study indicated that gut microbiota is a critical factor in the antidepressant effect of CSGS, which improve depression-related metabolic disturbance partly through gut microbiota.

## 1. Introduction

Depression is a range of neuro-behavioral disorders characterized by anhedonia, depressed mood, and high suicide rates [1,2]. The pathohistological hallmark of depression is neurotransmission decrease, hypothalamic–pituitary–adrenal (HPA) axis alterations, and immune system abnormalities [3]. Accumulating evidence by studying the gut microbiota diversity and its function from depression patients and animal models highlights the link between gut microbiota and depression [4,5]. Our recent study showed that the gut microbiota composition altered in chronic variable stress (CVS)-induced depression rats, in particular, two major phyla bacteria; the abundance of *Firmicutes* was reduced while the abundance of *Bacteroidetes* was increased, which was in line with other research findings in depressed patients [6,7]. Meanwhile, we also found that 11 genera bacteria were altered under the depression condition [7]. Taken together, these studies indicate that gut microbiota may causatively contribute to depression development and serve as a potential novel target for the prevention and the treatment of depression and other related diseases.

Indeed, emerging data suggest that the gut microbiota is a vital “organ” for the absorption and the metabolism of drugs. Meanwhile, drugs could regulate the composition of gut microbiota and further play a therapeutic effect, especially orally administered traditional Chinese medicine (TCM). For example, Gegen Qinlian decoction alleviates type 2 diabetes by enriching the amounts of beneficial bacteria, such as *Faecalibacterium* spp.; water extract of *Ganoderma lucidum* reduces obesity in mice by modulating *Firmicutes*-to-*Bacteroidetes* ratios and endotoxin-bearing *Proteobacteria* levels [8,9], and so forth. Chaihu-Shu-Gan-San (CSGS) consists of seven herbal medicines: Bupleuri Radix (*Bupleurum chinense* DC.), Citri Reticulatae Pericarpium (*Citrus reticulata* Blanco), Paeoniae Radix Alba (*Paeonia lactiflora* Pall.), Aurantii Fructus (*Citrus aurantium* L.), Cyperi Rhizoma (*Cyperus rotundus* L.), Chuanxiong Rhizoma (*Ligusticum chuanxiong* Hort.), and Glycyrrhizae Radix Et Rhizoma (*Glycyrrhiza uralensis* Fisch.), which has a long history of clinical use for treating of various gastrointestinal disorders and depression [10,11]. Our previous studies have already confirmed the antidepressant effect of CSGS based on metabolomics study. Specifically, CSGS treatment significantly improved ethology, neurotransmitters, and serum, urinary, and hippocampal metabolism disorders in CVS-induced depression rats [12,13].

However, the role of the gut microbiota in the pathogenesis of depression and the effect of CSGS decoction remain poorly unknown. Herein, an integrated gut microbiome and metabolome approach was performed based on the combination of CVS-induced depression and gnotobiotic rat models before and after CSGS treatment. This strategy provides more information on the interplay between gut microbiota and the antidepressant effect of CSGS.

## 2. Results

### 2.1. The Effect of CSGS against Depression-Like Behavioral Disorders Weakened after Antibiotic Treatment

**Figure 1:**
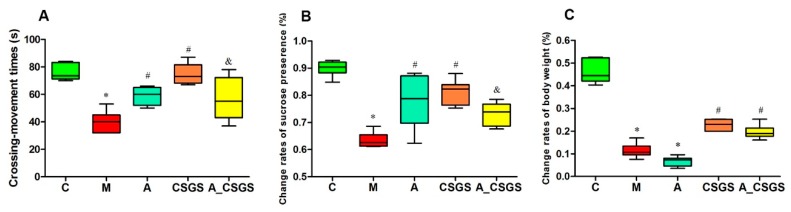
The results of behavioral tests and body weight in different treatment groups of rats: (**A**) the number of rearing and crossing; (**B**) the sucrose preference; (**C**) the body weight changes. C: control group; M: chronic variable stress (CVS) model group; A: Antibiotic treated group with CVS; CSGS: Chaihu-Shu-Gan-San (CSGS) treated group; A_CSGS: Antibiotic + CSGS treated group. * *p* < 0.05 vs. C; ^#^ *p* < 0.05 vs. M; ^&^ *p* < 0.05 vs. CSGS. The post hoc analysis of variance (ANOVA) used for statistical analysis and data with different superscript letters are significantly different.

The depression-like behaviors were evaluated by open-field and sucrose preference tests after treatment of CSGS and antibiotic combined CSGS treatment. As a result, the number of rearing and crossing and the sucrose preference in the CVS model group were decreased when compared with the control group. CSGS treatment significantly improved behavioral disorder, whereas the combined administration of antibiotic attenuated the effect of CSGS (Figure 1A,B). These results revealed that dysbiosis of the microbiota by antibiotic impacted behavioral disorder regulated by CSGS.

Instead, the rats from both CSGS groups with and without antibiotic treatment did not show difference in change rates of the bodyweight (Figure 1C). Thus, the regulation of CSGS on rat bodyweight was not affected with antibiotic administration.

### 2.2. The Effect of CSGS against Hippocampal Neurotransmitter Levels Alteration Weakened after Antibiotic Treatment

**Figure 2:**
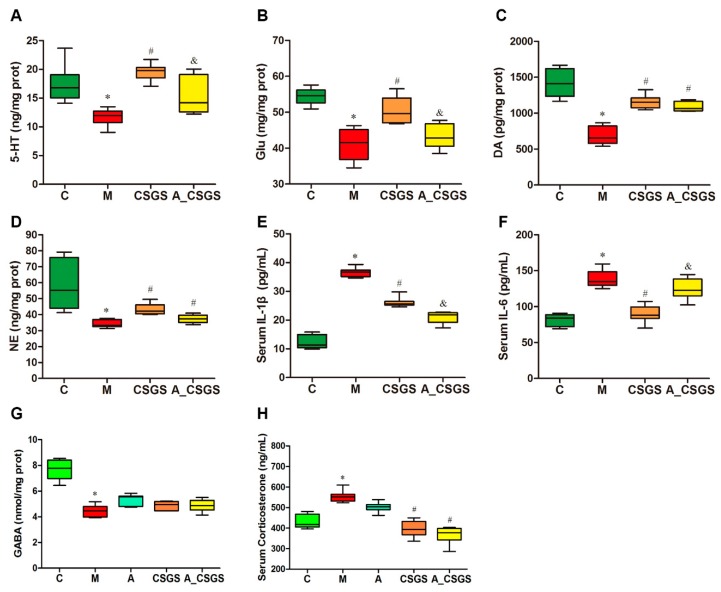
The levels of hippocampal 5-HT (**A**), glutamic acid (Glu) (**B**), dopamine (DA) (**C**), noradrenaline (NE) (**D**), gamma-aminobutyric acid (GABA) (**G**), and serum corticosterone (**H**), and inflammatory factors IL-1β (**E**) and IL-6 (**F**) levels in different treatment groups of rats. C: control group; M: CVS model group; A: Antibiotic treated group with CVS; CSGS: CSGS treated group; A_CSGS: Antibiotic + CSGS treated group. * *p* < 0.05 vs. C; ^#^ *p* < 0.05 vs. M; ^&^ *p* < 0.05 vs. CSGS. The post hoc ANOVA used for statistical analysis and data with different superscript letters are significantly different.

The hippocampus 5-HT, glutamic acid (Glu), dopamine (DA), noradrenaline (NE), and gamma-aminobutyric acid (GABA) levels were reduced under depression condition when compared with control rats. These reductions were totally normalized by the CSGS treatment, whereas the combined administration of antibiotic attenuated the effect of CSGS in the normalizing of 5-HT and Glu except for DA, NE, and GABA levels (Figure 2A–D,G). Serum corticosterone (CORT) and inflammatory factors IL-1β and IL-6 were all elevated in both the model and the antibiotic-induced group. This increase was totally blunted after CSGS treatment, whereas the antibiotic combined treatment weakened the ability of CSGS except for serum CORT (Figure 2E,F,H). These results suggest that antibiotic treatment attenuated the regulation effects of CSGS on the hippocampal neurotransmitter and the serum biochemistry in CVS-induced rats.

### 2.3. The Changes of Gut Microbiota in Depression Rats Treated with CSGS after Administration of Antibiotics

**Figure 3:**
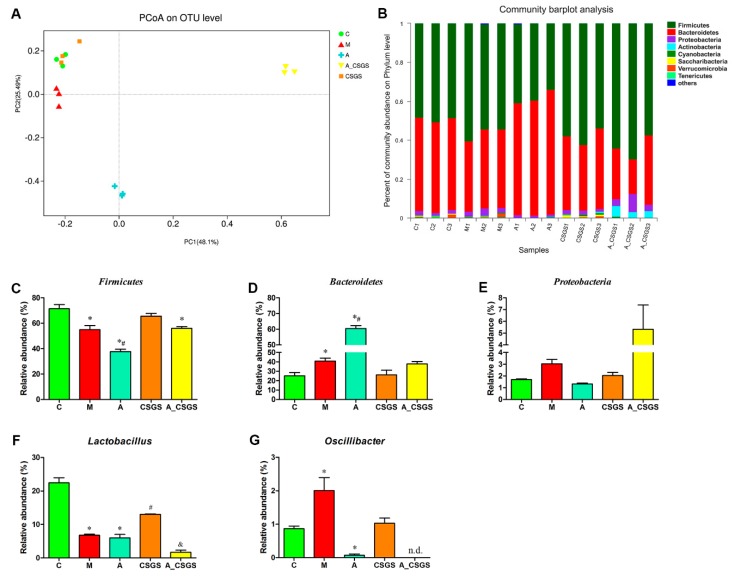
Principal coordinate analysis (PCoA) scatter plot of operational taxonomic units (OTUs) (**A**) and relative gut microbiota abundance at the phylum (**B**–**E**) and genus level (**F**,**G**) in different treatment groups of rats. C: control group; M: CVS model group; A: Antibiotic treated group with CVS; CSGS: CSGS treated group; A_CSGS: Antibiotic + CSGS treated group. * *p* < 0.05 vs. C; ^#^ *p* < 0.05 vs. M; ^&^ *p* < 0.05 vs. CSGS. n.d. indicates not detected. The post hoc ANOVA used for statistical analysis and data with different superscript letters are significantly different.

To analyze the fecal microbiota composition, the 16S rRNA gene sequencing-based method was performed. Principal coordinate analysis (PCoA) based on Bray–Curtis distance showed a clear separation of each experimental group. After CSGS treatment, the microbiota profile of rats (CSGS treated group) was close to the control group, which suggested dysbiosis gut microbiota was significantly improved based on operational taxonomic unit (OTU) level. Meanwhile, samples from A and A_CSGS groups were distinctly separated from C, M, and CSGS groups along the PCoA1 (PC1) (48.1%), which accounted for the largest principal component of all variables (Figure 3A). In addition, the alpha diversity of gut microbiota showed that OTUs from A and A_CSGS group samples were significantly reduced (Figure S1 and Table S1), which indicated that the diversity of gut microbiota decreased after antibiotic treatment. Furthermore, the jackknifed beta diversity and the hierarchical clustering analysis were carried out based on the unweighted pair group method with arithmetic mean (UPGMA) approach, and the result was in line with the PCoA (Figure 3B). Further analyses of the relative abundance of three major phyla (98% of the total bacterial)—*Firmicutes*, *Bacteroidetes*, and *Proteobacteria—*revealed a decrease in the abundance of *Firmicutes* and an increased abundance of *Bacteroidetes* in the model group compared with the normal group. However, there was a dramatic decrease in abundance of *Firmicutes*, and elevated *Bacteroidetes* were observed in the antibiotic, the combined antibiotic, and the CSGS treatment groups, while there was no significant difference at the phylum of *Proteobacteria* (Figure 3C–E). In addition, two higher relative abundances of genera, *Lactobacillus* and *Oscillibacter,* were analyzed. As a result, a decreased level of *Lactobacillus* and an increased level of *Oscillibacter* were improved by CSGS, whereas this good improvement effect of CSGS was suppressed after antibiotic treatment (A_CSGS vs. CSGS). Therefore, combined antibiotic and CSGS administration attenuated the improvement effect of CSGS on gut microbiota, especially the phylum level of *Firmicutes*.

### 2.4. Association of CSGS Effects with the Hippocampus Metabolites Related to Gut Microbiota

**Figure 4:**
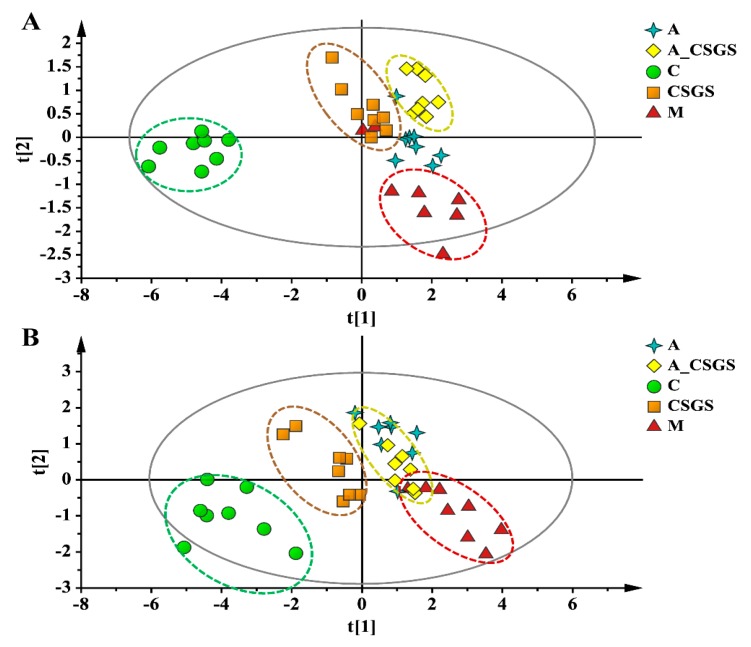
PCA score plot of hippocampus samples collected from different treatment groups of rats in (A, *R*^2^X = 0.761, *Q*^2^(cum) = 0.543) positive and (B, *R*^2^X = 0.831, *Q*^2^(cum) = 0.521) negative ion mode. C: control group; M: CVS model group; A: Antibiotic treated group with CVS; CSGS: CSGS treated group; A_CSGS: Antibiotic + CSGS treated group.

To describe the metabolic impacts of CSGS alone and combined antibiotic treatments, the metabolisms of hippocampus samples were assessed by using UPLC-Q-TOF/MS metabolomics approach (Figure S2). The principal component analysis (PCA) and orthogonal to partial least squares-discriminate analysis (OPLS-DA) model were performed to evaluate the metabolic patterns of each experimental group of rats after CVS, antibiotic, CSGS, and combined antibiotic CSGS treatments. The PCA scores plots collected in positive and in negative ion modes showed that the hippocampus tissues samples from the model group were distinctly separated from the normal group, and the CSGS treated group fairly differed from the model group but was similar to the control group. Meanwhile, the CSGS and the A_CSGS groups were separated by PCA1, whereas the A_CSGS group was far away from the control and the CSGS groups and closer to the model group after antibiotic treatment (Figure 4 and Figure S3). Accordingly, these results indicated that depression resulted in transparent alterations in hippocampus metabolic profile, and CSGS treatment dramatically altered the metabolic disorder but not in the antibiotic_CSGS treatment group. 

To further characterize the metabolites involved in the antidepressant effect of CSGS related to the gut microbiota, herein, we focused on comparing the significant difference of A vs. M group, CSGS vs. M group, and A_CSGS vs. M group. As a result, we found that the CSGS regulation effects of gamma-aminobutyric acid (H1), L-phenylalanine (H2), and pipecolic acid (H6) were not affected after further oral antibiotic treatment (A_CSGS vs. M); other metabolites of adenine (H7), myristic acid (H9), and 13-HDoHE (H12) were potential biomarkers related to depression, but CSGS could not prevent deviations of these metabolites (CSGS vs. M) (Table 1). Notably, two metabolites including the decreased 3-hydroxypicolinic acid (H4) and the increased inosine (H8) were markedly down-regulated after antibiotic treatment (A vs. M), which suggested that these metabolites-related bacteria were suppressed by the antibiotic. The CSGS ameliorated the abnormalities of H4 and H8 in the CVS group (CSGS vs. M) but not in the antibiotic_CVS group (A_CSGS vs. M). Therefore, H4 and H8 were considered the most associated metabolites with CSGS effect related to gut microbiota. 

## 3. Discussion

An integrated gut microbiome and metabolomic approach was performed to explore the impact of gut microbiota on the antidepressant effect of CSGS with and without antibiotic treatment. Our current results showed that gut microbiota is a critical factor in the antidepressant effect of CSGS, which involved in the improvement of CSGS on depression-like behavioral, neurotransmitters, serum corticosterone, inflammatory factor, and hippocampal metabolism of CVS rats.

Accumulating evidence highlights the potential association of gut microbiota with depression via the microbe–gut–brain axis [6,14,15]. Previous studies revealed altered gut microbiota composition in depression patients and in rodent models [6,7], and here, we confirmed these data in CVS-induced depression rats (Figure 3). Meanwhile, we also found that the disturbed gut microbiota composition was significantly improved at OTU level, as were dominant phyla *Firmicutes* and *Bacteroidetes* levels after CSGS treatment. However, treatment with the current regimen of antibiotic revealed a significant restructuring of the community composition in the A and the A_CSGS groups; in particular, PCoA scores from the sequences at OTU level showed that the A_CSGS group was far away from the C and the CSGS group. Specifically, there was a dramatic decrease in abundance of *Firmicutes* and increase in the abundance of *Bacteroidetes* were observed in the antibiotic, the combined antibiotic, and the CSGS treatment groups, while there was no significant difference at the phylum of *Proteobacteria*. Although we did not confirm whether the attenuated regulation effect of CSGS on gut microbiota dysfunction was due to modulating these abnormal bacterial phyla, our results provide further evidence that gut microbiota is an important factor affecting the antidepressant of CSGS.

Although the antidepressant mechanism of different responses to CSGS treatment has been extensively investigated, these studies are mainly focused on hippocampal 5-HT receptor and brain-derived neurotrophic factor (BDNF) either at pre- or post-treated CSGS that correlates with therapeutic outcomes [16,17,18]. Herein, we paid more attention to the overall hippocampal metabolism after CGSG and the combined oral administration broad-spectrum antibiotic treatment and then conducted analyses by the UPLC-Q-TOF/MS-based metabolomics approach. The results further validated our previous studies that CSGS reversed perturbations of the hippocampal metabolic phenotype and metabolites in CVS-induced depression rats [13]. Moreover, in this study, this good curative effect of CSGS was drastically blunted by the antibiotic in the A_CSGS group, and H4 and H8 were considered as the most associated metabolites with CSGS effects related to gut microbiota. H4 is the main metabolite of L-tryptophan catabolism, which is related to the development of depression [19]. It is also an important pyridine bacterial secondary metabolite [20]. In addition, H8 is an intermediate in the metabolism of adenosine, which plays a role in energy transfer as ATP and serves as a strong pro-inflammatory danger signal targeting specifically gram-positive bacteria [21]; meanwhile, H8 is a potential independent diagnostic biomarker for major depressive disorder (MDD) in children and adolescents [22]. Although we found the above results interesting, further study to explore the direct relationship of these two metabolites to CSGS effects related to gut microbiota is necessary. 

Moreover, there is ample evidence that the gut microbiota affects not only gastrointestinal physiology but also central nervous system (CNS) function and behavior via the microbe–gut–brain axis [3,23,24]. Behavioral tests such as open-field, sucrose preference, and forced swimming are necessary to evaluate the efficacy of antidepressant medicines. Validated oral CSGS extracts significantly prevented the abnormal ethological changes on rodent animal models [11]. In the current study, we investigated the effect of gut microbiota on the regulations of CSGS against depression-like etiological changes. The results indicated that the effect of CSGS was greatly blunted when combined with antibiotic treatment in the A_CSGS group. Furthermore, our previous study has established the linkage of altered gut microbiota and changed hippocampus catecholamine levels (5-HT, DA, and NE) based on Pearson’s correlation analysis [7]. Here, we further investigated the impact of gut microbiota on the mediation of CSGS for hippocampus catecholamine, serum corticosterone, and inflammatory factors. As a result, we found that the regulation of CSGS on 5-HT, Glu, IL-1β, and IL-6 attenuated if it was administrated with antibiotic together. However, there was no statistical difference of DA, NE, GABA, and serum corticosterone levels between the CSGS and the A_CSGS groups, indicating that these biochemical levels were not affected by antibiotic treatment.

In addition, increasing data suggest that gut microbiota plays a crucial role in TCM therapy by complicated interplay with TCM components [25]. Gut microbiota could transform TCM compounds into metabolites, while TCM chemicals could improve the composition and the functions of gut microbiota. As one of the TCMs, when CSGS is given orally, the interactions between compounds in CSGS and gut microbiota are unavoidable [25]. Indeed, there are some reports showing that the major active compounds in CSGS are metabolized by gut microbiota; for example, albiflorin is metabolized by gut microbiota and transformed to benzoic acid crossing the blood–brain barrier [26]. Therefore, further study to focus on the interactions between major compounds in CSGS and specific gut microbiota genera/species is necessary.

In conclusion, our results suggest that gut microbiota dysbiosis after antibiotic treatment attenuated the antidepressant effect of CSGS in CVS-induced depression rats. These data also indicate that the gnotobiotic model (antibiotic-induced) is useful for assessing the role of gut microbiota and its interactions with medications.

## 4. Materials and Methods 

### 4.1. Reagents

Methanol, acetonitrile (J. T. Baker, Phillipsburg, NJ, USA), purified water (18.2 MΩ), and formic acid (Sigma-Aldrich, St. Louis, MO, USA) were of HPLC grade. Leucine-enkephalin was obtained from Sigma-Aldrich (St. Louis, MO, USA). Imipenem/cilastatin sodium (broad spectrum *β*-lactam antibiotic), named Tienam, was purchased from MSD Pharmaceutical Company Limited (Hangzhou, China). The preparation and the quality control method of CSGS extract was consistent with the previous reports [13].

### 4.2. Animal Experiments

All rat experimental procedures were approved by the Ethics Committee of the Institute of Medicinal Plant Development, CAMS & PUMC. Forty male Wistar rats with a body weight of 200 ± 20 g were obtained from the Beijing Vital River Laboratory Animal Technology Co., Ltd. (Beijing, China). All animals were housed in an Specific pathogen Free (SPF) animal facility (temperature, 20–25 °C; relative humidity, 40–60%; light-dark cycles, 12 h–12 h). Until required for experiments, the rats were provided with a standard commercial diet and tap water ad libitum. The rats were randomly segregated into five groups (*n* = 8 per group): (1) control group (C); (2) CVS model group (M); (3) antibiotic treatment group with CVS (A), oral administration dosed with antibiotic imipenem/cilastatin sodium at a daily dose of 75 mg kg^−1^ of body weight from day 25 to day 28 [27]; (4) CSGS treated group with CVS (CSGS), where rats received CSGS water extracts by oral administration at the dose of 7.0 g·kg^−1^ once daily for 28 days; (5) antibiotic_CSGS group with CVS (A_CSGS), where rats received CSGS water extracts at the dose of 7.0 g·kg^−1^ once daily for 28 days. Meanwhile, rats received the same dose of antibiotic imipenem/cilastatin sodium from day 25 to day 28. All rats except those in the control group were subjected to a series of variable stimuli as previously described [11]. 

### 4.3. Sample Collection and Preparation

Fecal samples (2 pellets) were collected from each rat at the end of the CVS experiment (day 28); the samples were placed in sterile conical tubes and immediately stored at −80 °C for microbial community analysis. After the behavior test (day 30), blood was collected in a standard protocol under urethane anesthesia by intraperitoneal injection, and then serum sample was obtained and kept at −80 °C for later analysis. Serum for corticosterone and inflammatory factors were examined with the ELISA kit (MP Biomedicals, Santa Ana, CA, USA). Brains were removed, and the hippocampus regions were dissected and immediately transferred into liquid nitrogen to be frozen. Hippocampus samples were used for serotonin (5-HT), glutamic acid (Glu), dopamine (DA), noradrenaline (NE), and gamma-aminobutyric acid (GABA) analysis with ELISA kit (MP Biomedicals, Santa Ana, CA, USA) and metabolomics study. Approximately 50 mg of hippocampus tissues samples were weighed and homogenized using 1 mL of 50% ice-cold methanol, and a protein-free supernatant was obtained. Following, the supernatant was transferred to a new tube and blow-dried under nitrogen, and then reconstituted with 100 μL of acetonitrile-water (10: 90, *v*/*v*) for UPLC-MS analysis. 

### 4.4. Gut Microbiota Analysis

The fecal microbiome measurement and pretreatment were based on our previous study [7]. Briefly, fecal total DNA was extracted using the E.Z.N.A.^®^ Soil DNA Kit (Omega Bio-Tek, Norcross, GA, USA). Gut microbiota 16S rRNA (V3-V4) of all fecal samples was amplified and measured using the IlluminaMiseq platform. The 16S rRNA gene sequences data were analyzed using the QIIME software package. Operational taxonomic units (OTUs) were clustered using UPARSE (version 7.1, http://drive5.com/uparse/) according to representative sequences based on Usearch [28] and classified against the Greengenes Database [29] with a threshold of 97% sequence similarity. Statistical analysis of Bray–Curtis dissimilarities were calculated using the relative abundance of bacterial genera performed using R (version 3.2.1) and the adonis function in the R package “vegan”.

### 4.5. Metabolic Profiling

Chromatographic analysis was performed in a Waters Acquity^TM^ Ultra Performance LC system (Waters Corp., Milford, MA, USA). For hippocampus tissues analysis, an HSS T3 column (2.1 × 100 mm, 1.8 μm, Waters Corp., Milford, MA, USA) was performed. The column was maintained 4 °C, and a 5 μL injection volume was used for all cases. A flow rate of 0.40 mL/min was set. The mobile phase and the linear gradient programs for all samples are listed as follows: solvents A (acetonitrile + 0.1% formic acid), B (water + 0.1% formic acid); the gradient program: 0–0.5 min, 1% B; 0.5–4 min, 1–60% B; 4–10 min, 60–99% B; 10–13 min, washing with 100% B, and 13–15 min, equilibration with 1% B. The eluent from the column was directed to the mass spectrometer without split.

Mass spectral data were acquired using a Waters SYNAPT G2 HDMS (Waters Corp., Manchester, UK) Q-TOF mass spectrometer equipped with an electrospray ionization source (ESI) operating in positive and negative ion scan modes. The optimized parameters of TOF MS were set as previously described [7]. Continuum mode data were collected, and the mass range was set at *m*/*z* 50 to *m*/*z* 1200.

### 4.6. Data Processing and Statistical Analysis

The MS data (.raw) from each group of hippocampus tissues samples were first processed using MarkerLynx software (Waters Corp., Manchester, UK; version 4.1). This process provides a list of retention times and mass pairs with corresponding intensities of each metabolite from every sample in the data set. 

The above data set was imported into the software of SIMCA-P (version13.0, Umetric, Umea, Sweden) and further analyzed by the multiple statistic methods of principal component analysis (PCA) and orthogonal to partial least squares-discriminate analysis (OPLS-DA). The PCA method was applied to investigate whether outliers exist. OPLS-DA was employed to pick out discriminating variables contributing to the classification among the experimental samples. In the OPLS-DA model, the variable importance of project (VIP) value, coefficient plot, and S-plot were used as the standard to select potential pharmacodynamic biomarkers responsible for CSGS treatment.

One-way ANOVA was performed for statistical analysis using the Statistical Package for Social Science program (SPSS 16.0, Chicago, IL, USA) and R (version 3.2.1). The significance threshold was set as *p* < 0.05 being considered significant.

## Figures and Tables

**Table 1:** Hippocampus differential metabolites based on UPLC-Q-TOF/MS.

No.	Metabolites	RT (min)	*m*/*z*	Adduction	Delta (mDa)	M vs. C	A vs. M	CSGS vs. M	A_CSGS vs. M
H1	Gamma-Aminobutyric acid ^ES-^	0.58	124.0282	[M + Na − 2H]^−^	<0.01	↓ ^b^	↑ ^a^	↑ ^b^	↑ ^b^
H2	l-Phenylalanine ^ES−^	0.59	146.0665	[M + H_2_O − H]^−^	0.01	↓ ^b^	↑ ^a^	↑ ^b^	↑ ^b^
H3	Glycine ^ES+^	0.75	76.0217	[M + H]^+^	0.02	↓ ^b^	—	↑ ^b^	—
H4	3-Hydroxypicolinic acid ^ES+^	0.76	162.0220	[M + Na]^+^	0.01	↓ ^b^	↓ ^b^	↑ ^a^	—
H5	Cysteinyl-Tryptophan ^ES−^	0.79	306.0925	[M − H]^-^	<0.01	↓ ^b^	—	↑ ^b^	—
H6	Pipecolic acid ^ES−^	1.24	128.0562	[M − H]^−^	0.02	↓ ^b^	—	↑ ^b^	↑ ^b^
H7	Adenine ^ES+^	1.25	136.0616	[M+H]^+^	<0.01	↓ ^b^	↑ ^a^	—	—
H8	Inosine ^ES−^	1.28	267.0915	[M − H]^−^	0.02	↑ ^b^	↓ ^b^	↓ ^b^	—
H9	Myristic acid ^ES+^	3.51	246.2427	[M + NH_4_]^+^	<0.01	↑ ^b^	—	—	—
H10	Tetradecanoylcarnitine ^ES+^	4.89	372.3101	[M + H]^+^	<0.01	↓ ^b^	—	↑ ^a^	—
H11	l-Lactic acid ^ES+^	5.00	91.0540	[M + H]^+^	0.02	↑ ^b^	—	↓ ^a^	—
H12	13-HDoHE ^ES+^	5.68	332.3308	[M + NH_4_]^+^	0.04	↑ ^b^	↓ ^a^	—	—
H13	LysoPC(16:0) ^ES+^	5.95	496.3401	[M + H]^+^	<0.01	↑ ^b^	—	↓ ^a^	—

↑: up-regulated, ↓: down-regulated; ^a^ *p* < 0.05, ^b^ *p* < 0.01. —: no significant differences.

## Supplementary Materials

The following are available online at https://www.mdpi.com/2218-1989/10/2/63/s1, Figure S1: Alpha diversity plot of OTUs feces microbiota community in different treatment groups of rats; Figure S2: Typical UPLC-Q-TOF/MS base peak intensity (BPI) chromatograms of hippocampus samples from the control group and model group in positive ion mode (A and B) and in negative ion mode (C and D); Figure S3: OPLS-DA and S-Plot score plot of hippocampus samples collected from different treatment groups of rats in (A and B, *R*^2^X = 0.468, *R*^2^Y = 1, *Q*^2^ (cum) = 0.743) positive and (C and D, *R*^2^X = 0.57, *R*^2^Y = 0.999, *Q*^2^ (cum) = 0.859) negative ion mode. Table S1: the sequencing number of each experimental group samples.

## Abbreviations

CSGS	Chaihu-Shu-Gan-San
CVS	chronic variable stress
TCM	Traditional Chinese medicine
OTU	Operational taxonomic unit
ESI	electrospray ionization source
PCA	principal component analysis
OPLS-DA	orthogonal to partial least squares-discriminate analysis
VIP	variable importance of project
PCoA	principal coordinate analysis

## References

[1] Paykel E.S. (2006). Depression: Major problem for public health. Epidemiologia e Psichiatria Sociale.

[2] Faulconbridge L.F., Wadden T.A., Berkowitz R.I., Sarwer D.B., Womble L.G., Hesson L.A., Stunkard A.J., Fabricatore A.N. (2009). Changes in symptoms of depression with weight loss: Results of a randomized trial. Obesity.

[3] Zheng P., Zeng B., Zhou C., Liu M., Fang Z., Xu X., Zeng L., Chen J., Fan S., Du X. (2016). Gut microbiome remodeling induces depressive-like behaviors through a pathway mediated by the host’s metabolism. Mol. Psychiatry.

[4] Kelly J.R., Borre Y., C O.B., Patterson E., El Aidy S., Deane J., Kennedy P.J., Beers S., Scott K., Moloney G. (2016). Transferring the blues: Depression-associated gut microbiota induces neurobehavioural changes in the rat. J. Psychiatr. Res..

[5] Cepeda M.S., Katz E.G., Blacketer C. (2017). Microbiome-Gut-Brain Axis: Probiotics and Their Association with Depression. J. Neuropsychiatry Clin. Neurosci..

[6] Jiang H., Ling Z., Zhang Y., Mao H., Ma Z., Yin Y., Wang W., Tang W., Tan Z., Shi J. (2015). Altered fecal microbiota composition in patients with major depressive disorder. Brain Behav. Immun..

[7] Yu M., Jia H., Zhou C., Yang Y., Zhao Y., Yang M., Zou Z. (2017). Variations in gut microbiota and fecal metabolic phenotype associated with depression by 16S rRNA gene sequencing and LC/MS-based metabolomics. J. Pharm. Biomed. Anal..

[8] Xu J., Lian F., Zhao L., Zhao Y., Chen X., Zhang X., Guo Y., Zhang C., Zhou Q., Xue Z. (2015). Structural modulation of gut microbiota during alleviation of type 2 diabetes with a Chinese herbal formula. ISME J..

[9] Chang C.J., Lin C.S., Lu C.C., Martel J., Ko Y.F., Ojcius D.M., Tseng S.F., Wu T.R., Chen Y.Y., Young J.D. (2015). Ganoderma lucidum reduces obesity in mice by modulating the composition of the gut microbiota. Nat. Commun..

[10] Qin F., Liu J.Y., Yuan J.H. (2013). Chaihu-Shugan-San, an oriental herbal preparation, for the treatment of chronic gastritis: A meta-analysis of randomized controlled trials. J. Ethnopharmacol..

[11] Su Z.H., Li S.Q., Zou G.A., Yu C.Y., Sun Y.G., Zhang H.W., Gu Y., Zou Z.M. (2011). Urinary metabonomics study of anti-depressive effect of Chaihu-Shu-Gan-San on an experimental model of depression induced by chronic variable stress in rats. J. Pharm. Biomed. Anal..

[12] Jia H.M., Yu M., Ma L.Y., Zhang H.W., Zou Z.M. (2017). Chaihu-Shu-Gan-San regulates phospholipids and bile acid metabolism against hepatic injury induced by chronic unpredictable stress in rat. J. Chromatogr. B Anal. Technol. Biomed. Life Sci..

[13] Su Z.H., Jia H.M., Zhang H.W., Feng Y.F., An L., Zou Z.M. (2014). Hippocampus and serum metabolomic studies to explore the regulation of Chaihu-Shu-Gan-San on metabolic network disturbances of rats exposed to chronic variable stress. Mol. Biosyst..

[14] Sharon G., Sampson T.R., Geschwind D.H., Mazmanian S.K. (2016). The Central Nervous System and the Gut Microbiome. Cell.

[15] Li B., Guo K., Zeng L., Zeng B., Huo R., Luo Y., Wang H., Dong M., Zheng P., Zhou C. (2018). Metabolite identification in fecal microbiota transplantation mouse livers and combined proteomics with chronic unpredictive mild stress mouse livers. Transl. Psychiatry.

[16] Sun Y., Xu X., Zhang J., Chen Y. (2018). Treatment of depression with Chai Hu Shu Gan San: A systematic review and meta-analysis of 42 randomized controlled trials. BMC Complement. Altern. Med..

[17] Yang P., Li L., Liu X.J., Cai X., Sun M.Z., He J.F., Zeng G., Huang H.Y. (2016). Effect of Chaihu-Shugan-San on the mRNA expression of the 5-HT1A receptor and cellular proliferation in the hippocampus of epileptic rats with depression. Exp. Ther. Med..

[18] Chen X.Q., Li C.F., Chen S.J., Liang W.N., Wang M., Wang S.S., Dong S.Q., Yi L.T., Li C.D. (2018). The antidepressant-like effects of Chaihu Shugan San: Dependent on the hippocampal BDNF-TrkB-ERK/Akt signaling activation in perimenopausal depression-like rats. Biomed. Pharmacother..

[19] Dehhaghi M., Kazemi Shariat Panahi H., Guillemin G.J. (2019). Microorganisms, Tryptophan Metabolism, and Kynurenine Pathway: A Complex Interconnected Loop Influencing Human Health Status. IJTR.

[20] Yun X., Zhang Q., Lv M., Deng H., Deng Z., Yu Y. (2019). In vitro reconstitution of the biosynthetic pathway of 3-hydroxypicolinic acid. Org. Biomol. Chem..

[21] Zhou X., Liu L., Lan X., Cohen D., Zhang Y., Ravindran A.V., Yuan S., Zheng P., Coghill D., Yang L. (2018). Polyunsaturated fatty acids metabolism, purine metabolism and inosine as potential independent diagnostic biomarkers for major depressive disorder in children and adolescents. Mol. Psychiatry.

[22] Lalles J.P. (2016). Microbiota-host interplay at the gut epithelial level, health and nutrition. J. Anim. Sci. Biotechnol..

[23] Sampson T.R., Debelius J.W., Thron T., Janssen S., Shastri G.G., Ilhan Z.E., Challis C., Schretter C.E., Rocha S., Gradinaru V. (2016). Gut Microbiota Regulate Motor Deficits and Neuroinflammation in a Model of Parkinson’s Disease. Cell.

[24] Kang D.W., Adams J.B., Gregory A.C., Borody T., Chittick L., Fasano A., Khoruts A., Geis E., Maldonado J., McDonough-Means S. (2017). Microbiota Transfer Therapy alters gut ecosystem and improves gastrointestinal and autism symptoms: An open-label study. Microbiome.

[25] Xu J., Chen H.B., Li S.L. (2017). Understanding the Molecular Mechanisms of the Interplay Between Herbal Medicines and Gut Microbiota. Med. Res. Rev..

[26] Zhao Z.X., Fu J., Ma S.R., Peng R., Yu J.B., Cong L., Pan L.B., Zhang Z.G., Tian H., Che C.T. (2018). Gut-brain axis metabolic pathway regulates antidepressant efficacy of albiflorin. Theranostics.

[27] Zheng X., Xie G., Zhao A., Zhao L., Yao C., Chiu N.H., Zhou Z., Bao Y., Jia W., Nicholson J.K. (2011). The footprints of gut microbial-mammalian co-metabolism. J. Proteome Res..

[28] Hartmann M., Howes C.G., VanInsberghe D., Yu H., Bachar D., Christen R., Henrik Nilsson R., Hallam S.J., Mohn W.W. (2012). Significant and persistent impact of timber harvesting on soil microbial communities in Northern coniferous forests. ISME J..

[29] DeSantis T.Z., Hugenholtz P., Larsen N., Rojas M., Brodie E.L., Keller K., Huber T., Dalevi D., Hu P., Andersen G.L. (2006). Greengenes, a chimera-checked 16S rRNA gene database and workbench compatible with ARB. Appl. Environ. Microbiol..

