# Supplementary material for: Gut Microbiota Is the Key to the Antidepressant Effect of Chaihu-Shu-Gan-San
Source: Metabolites. 2020 Feb 10;10(2):63. doi: 10.3390/metabo10020063 (PMC7074034; doi:10.3390/metabo10020063)
Supplement: Supplementary file 1 [file metabolites-10-00063-s001.pdf]

The CVS procedures as follows: (1) immobilization for 5h, (2) swimming in 15 ◦ C water for 5min, (3) food withholding for 48h, (4) thermal stimulus in 45 ◦ C water for 5min, (5) water intake forbidden for 48h, (6) electric shock to pelma (electric current for 1mA, 2s per shock, 2 shocks per minute), (7) noise stimulus at 110dB, and (8) stroboflash—2 flashs per second for 4h. These stimuli were presented in an unpredictable manner with one or two of these on each rat each day. Each stimulus was performed at least 3 times in 28 days.

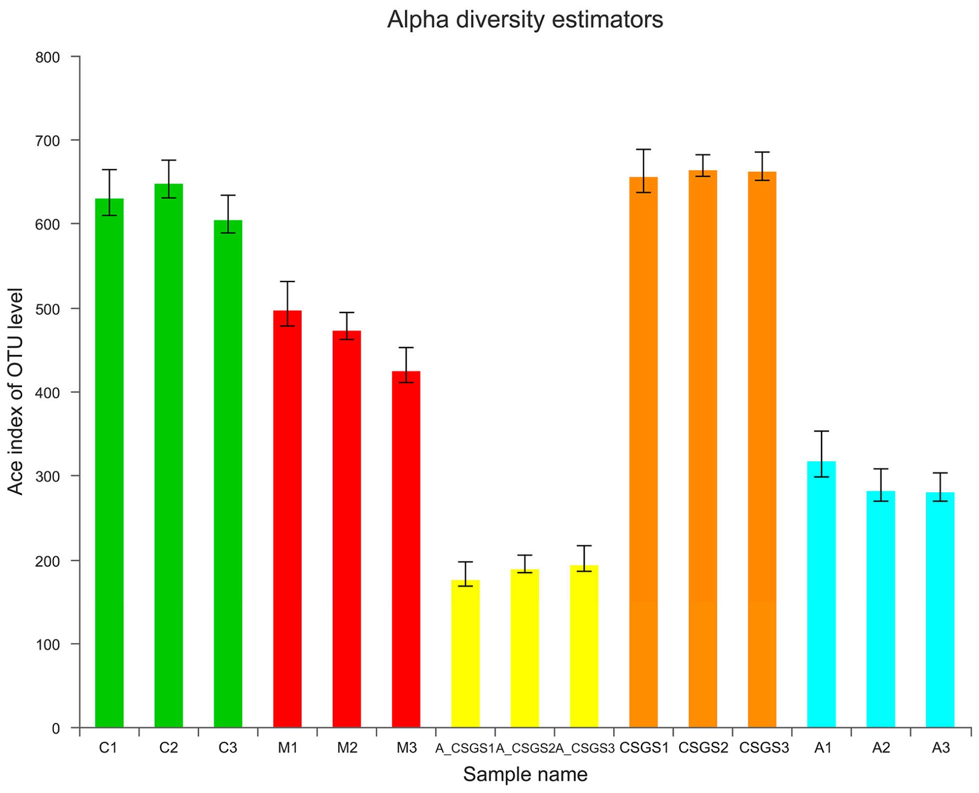

Figure S1. Alpha diversity plot of OTUs feces microbiota community in different treatment groups of rats.

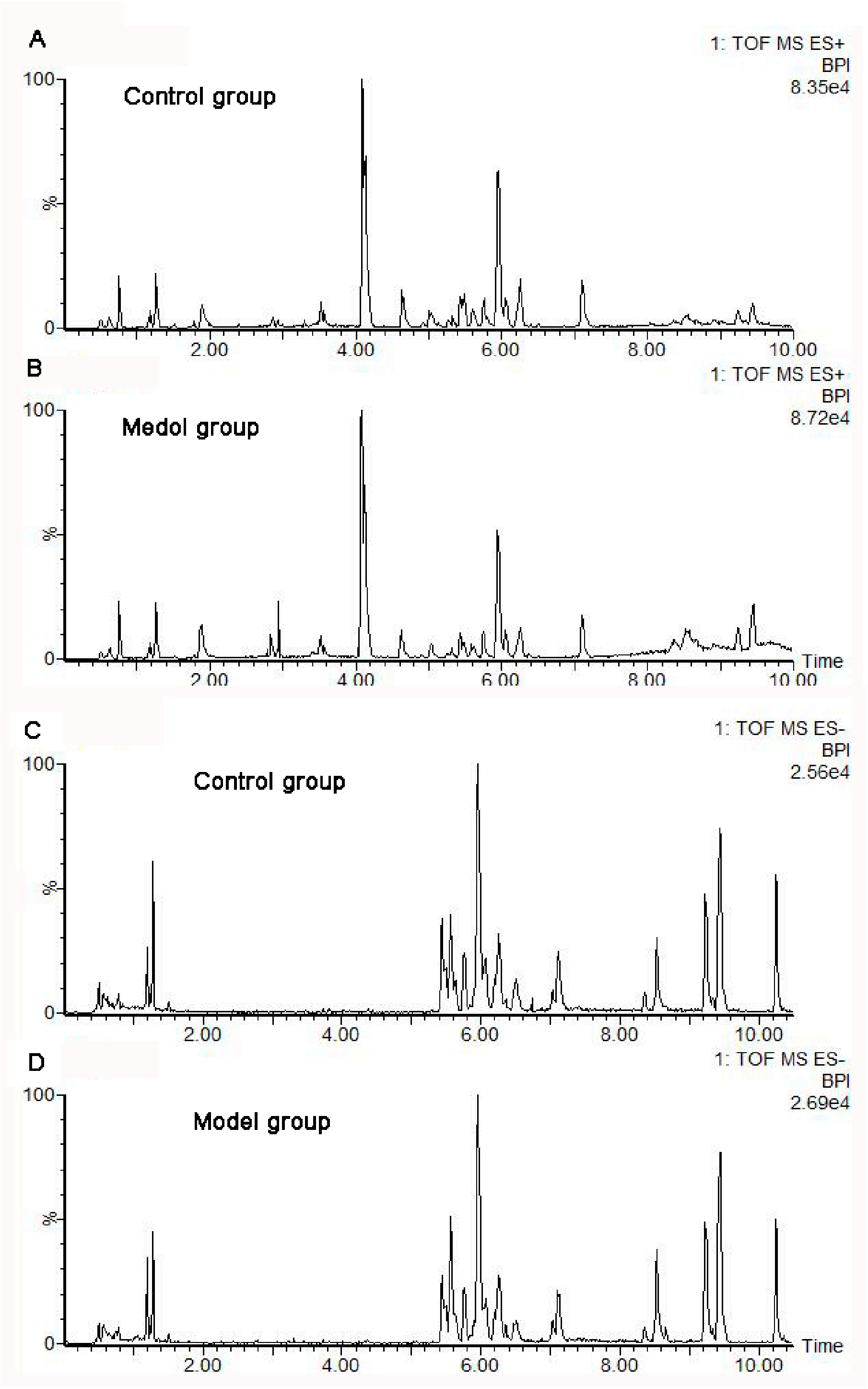

Figure S2. Typical UPLC-Q-TOF/MS base peak intensity (BPI) chromatograms of hippocampus samples from the control group and model group in positive ion mode (A and B) and in negative ion mode (C and D).

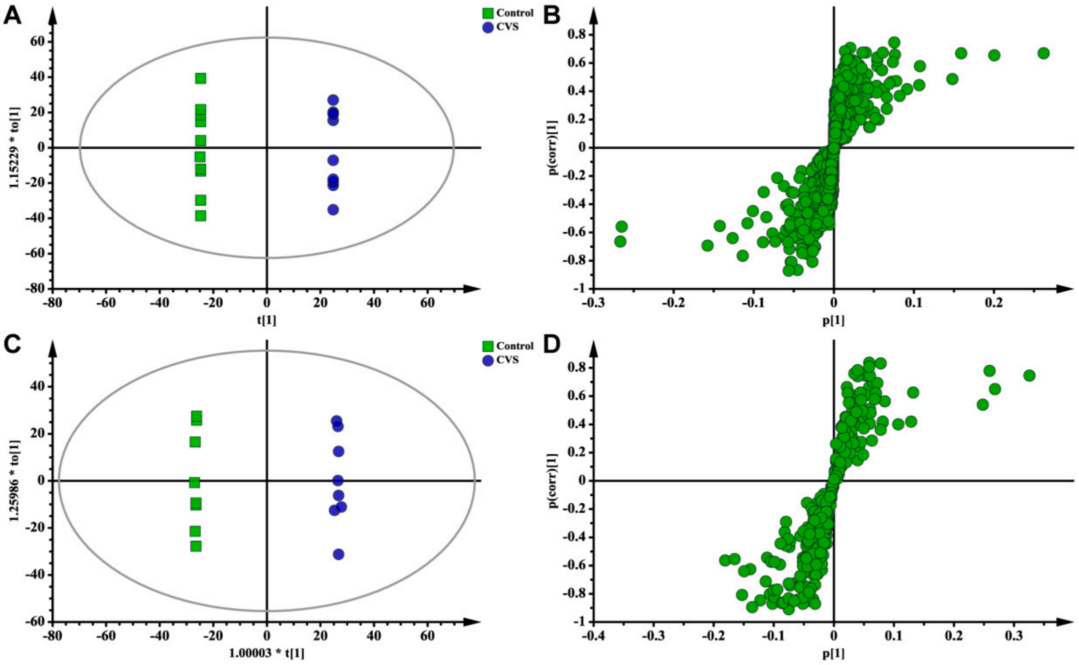

Figure S3. OPLS-DA and S-Plot score plot of hippocampus samples collected from different treatment groups of rats in (A and B, *R2X=0.468, R2Y=1, Q2 (cum)=0.743*) positive and (C and D, R2X=0.57, R2Y=0.999, Q2 (cum)=0.859) negative ion mode.

Table S1 the sequencing number of each experimental group samples

Sample\Information	Seq_number
C1	52986
C2	57182
C3	30872
M1	29550
M2	32606
M3	30656
CSGS1	43242
CSGS2	125702
CSGS3	48738
A_CSGS1	23504
A_CSGS2	33888
A_CSGS3	64957
A1	26502
A2	33920
A3	27807
